# The Positive Pharmacy Care Law revisited: an area-level analysis of the relationship between community pharmacy distribution, urbanicity and deprivation in England

**DOI:** 10.1136/bmjopen-2024-095540

**Published:** 2025-05-11

**Authors:** Eman Zied Abozied, Luke Aaron Munford, Alison Copeland, Adetayo Kasim, Andy Husband, Clare Bambra, Adam Todd

**Affiliations:** 1 Population Health Sciences Institute, Newcastle University Faculty of Medical Sciences, Newcastle upon Tyne, UK; 2 Division of Population Health, Health Services Research and Primary Care, The University of Manchester, Manchester, UK; 3 NIHR Applied Research Collaboration Greater Manchester (ARC-GM), Manchester, UK; 4 Newcastle University School of Geography, Politics and Sociology, Newcastle upon Tyne, UK; 5 Newcastle University, Newcastle upon Tyne, UK; 6 Research Statistics, GlaxoSmithKline, London, UK; 7 School of Pharmacy, Newcastle University, Newcastle upon Tyne, UK; 8 Newcastle NIHR Patient Safety Research Collaboration, Newcastle University, Newcastle upon Tyne, UK

**Keywords:** Health Services, PUBLIC HEALTH, Pharmacists

## Abstract

**Abstract:**

**Objectives:**

(1) Determine geographical access to community pharmacy in England, (2) explore the relationship between community pharmacy access and urbanity and multiple deprivation and (3) understand any changes in access over time.

**Design:**

An area-level spatial analysis study exploring the relationship between spatial access to and availability of community pharmacies over the past 10 years from 2014 to 2023, deprivation and urbanicity, using Geographic Information System and descriptive statistics on a Middle layer Super Output Area level.

**Primary outcome measure:**

Availability per 10 000 people of a community pharmacy in their local area.

**Results:**

For geographical access, in 2014, 91.3% of people lived within a 20-minute walk to a community pharmacy and, in 2023, this number increased to 91.7%. There was a positive relationship between geographical community pharmacy access and urbanity and geographical community pharmacy access and deprivation. For availability, the median number of community pharmacies per 10 000 people in 2014 was 1.60, while in 2023, the number reduced to 1.51 community pharmacies per 10 000 people. The most deprived areas were more likely to lose a pharmacy, compared with the least deprived areas (OR 1.65 (1.38, 1.98)).

**Conclusions:**

There is high access to community pharmacies in England with access to a community pharmacy greatest in the most deprived areas, showing that the ‘positive pharmacy care law’ remains. However, the ‘positive pharmacy care law’ is eroding as the availability of community pharmacies has reduced over time—particularly in deprived areas, with more people reliant on each community pharmacy.

**STRENGTHS AND LIMITATIONS OF THIS STUDY**

- Community pharmacy access was conceptualised in two ways to enable insights into both geographical accessibility and population-wide availability.
- Middle layer Super Output Areas were used as a better conceptual representation of a person’s local area or town than a Lower layer Super Output Area.
- We acknowledge the limitation that just because a person lives within 20-minute walking distance (1 mile) of a community pharmacy does not mean they can walk the distance due to their own abilities and other factors.
- Midyear population estimates were not yet available after 2020, so this may lead to the overestimation of the community pharmacies per 10 000 people for years post 2020 as the real population denominator may have increased.

## Background

 Community pharmacies are an important component of healthcare systems around the world and deliver a range of public health and clinical services to the general public. Examples of such services include smoking cessation, emergency hormonal contraception, hypertension screening and ‘influenza’ vaccination programmes.^1 2^ Recent policy developments have also seen the commissioning of clinical pharmacy services to manage various common conditions. For example, in England, through a ‘Pharmacy First’ scheme, community pharmacies are now able to treat seven common conditions following a defined clinical pathway.^3^ In 2014, we established that, in England, community pharmacies followed a ‘positive pharmacy care law’^4^ whereby the availability of community pharmacies was greatest in the most deprived communities (defined through area-level multiple deprivation). This was in contrast to the well-established ‘inverse care law’ for other healthcare services in England and internationally, whereby there is less provision in the most deprived areas even though they have the highest health needs (ie, a higher morbidity burden).^5^ In related analysis, we found that more people lived within a 20-minute walk to community pharmacies than other primary care providers, including general practice.^6^ The positive pharmacy care supported the idea of using community pharmacies as a way to promote health and well-being interventions to local communities.^7^

Since our discovery of the ‘positive pharmacy care law’ 10 years ago, there have been funding cuts across the sector that have led to community pharmacy closures—with some estimations suggesting that more than 1000 community pharmacies in England have closed in this time.^8^ Given the changing landscape of the community pharmacy network, it is important to understand if the positive pharmacy care law is still in operation, as this could have implications for the commissioning of future services. This work, therefore, seeks to address this knowledge gap and aims to (i) determine geographical access to community pharmacy in England, (ii) explore the relationship between community pharmacy access and urbanity and multiple deprivation and (iii) understand any changes in access over time.

## Methods

### Study design

This study explores the relationship between spatial access to and availability of community pharmacies over the past 10 years from 2014 to 2023, deprivation and urbanicity, using Geographic Information System and statistical analysis on a Middle layer Super Output Area (MSOA level).

### Definitions

#### Community pharmacy

Registered with the General Pharmaceutical Council *as premises for the compounding, procurement, storage and distribution of medicines and appliances*. In this study, we excluded ‘internet only’ pharmacies and hospital pharmacies as they are not accessible to the general community.

#### Middle layer Super Output Areas

A geographical area defined by the Office for National Statistics which has a minimum size of 5000 residents and 2000 households with an average population size of 7800. They fit within local authority boundaries and are generally analogous to a small town.^9^

#### Urban/rural classification

An index of eight main categories—major conurbation, minor conurbation, cities and towns, towns and fringe, and rural villages and hamlets which we consolidated to four categories (online supplemental table S1). Every settlement with a population over 10 000 is urban and every settlement below that is rural. Settlements are further classified using their building and population density and surrounding setting.^10^

#### Index of Multiple Deprivation (IMD) quintile

The IMD is a measure of relative deprivation for small geographic areas of the UK. Each small area is ranked based on seven domains of deprivation (income, employment, education, health, crime, barriers to housing and services, and living environment). IMD is split into five quintiles based on relative disadvantage, with quintile 1 being the most deprived and quintile 5 being the least deprived.^11^

### Outcome

We used three measures to assess a person’s access to a community pharmacy:

1. Community pharmacy availability per 10 000 people (number of pharmacies in an MSOA × 10 000/population of the MSOA).
2. Community pharmacy access: whether or not the straight-line distance is within a 20-minute walk from the population weighted centroid of the MSOA (where most people live)^12^ to the nearest pharmacy, approximately 1 mile or 1.6 km (1=yes, 0=no).
3. Community pharmacy change over time: whether or not a community has lost a pharmacy between 2023 and 2014 based on the availability measure. A negative value means there is a loss, which is then operationalised as a binary variable (0=gain or no change, 1=loss).

### Data sources

We used the 2011 MSOA boundaries as a base map because the 2019 IMD is based on them. We used the Ordnance Survey (OS) Points of Interest datasets to find the locations of community pharmacies, from the September release of each year from 2014 to 2023. The data were downloaded from Edina Digimap using an Educational Use licence.^13^,^22^ Additional data were attached to the initial base map to enable statistical analysis. We used the IMD 2019 on MSOA level compiled by mySociety,^23^ which also contains the region of each MSOA, the Rural Urban Classification (2011) of MSOAs in England from the Office for National Statistics,^24^ and the midyear population estimates for 2014–2020 for MSOAs^25^ to calculate the availability per 10 000 people measure. To calculate the distance to the nearest pharmacy, we used the population-weighted centroid of each MSOA as an origin point, which corresponds to the coordinates where the majority of people live within the MSOA.^26^ For the age and ethnicity structure at the first time point, we used data from the 2011 census from the NOMIS database.^27 28^

### Geographical data workflow

#### Base map

The MSOA boundaries were linked to IMD, urban/rural classification, region and MSOA population estimate in Quantum Geographic Information System (QGIS) Software^29^ using a spatial join by MSOA ID code.

#### Data cleaning

Initial investigations found a discrepancy between the number of registered pharmacies listed by the General Pharmaceutical Council and community pharmacies present in the OS dataset. The General Pharmaceutical Council is responsible for the regulation of pharmacists, pharmacy technicians and pharmacy premises in England, Scotland and Wales. In the OS dataset, we found an undercount of pharmacies from 2014 to 2019 then an overcount from 2020 onwards. The overcount was due to hospital pharmacies included in the OS dataset and classified as community pharmacies. The data was cleaned and filtered to remove online-only pharmacies and hospital pharmacies and compared with the General Pharmaceutical Council registered pharmacy list to make sure the number of pharmacies OS data was not greater than the General Pharmaceutical Council list. The data cleaning procedure is expanded on in online supplemental tables S7 and S8.

#### Spatial analysis

Two spatial analyses were undertaken in QGIS for each year of the dataset from 2014 to 2023:

1. The algorithm ‘count points in polygon’ to find the number of pharmacies (represented as points) within the MSOA boundary polygon.
2. Straight line distance from population-weighted centroid (the area where most people live) to the nearest pharmacy.

The results of these analyses were linked to the base map and exported from QGIS to Stata.^30^

### Statistical analysis

Two measures were generated in Stata, the availability per 10 000 people and whether there was a community pharmacy within a 20-minute walk. The number of community pharmacies in an MSOA was divided by the population of that MSOA to determine the availability per 10 000 people. The population denominator changes year on year to reflect population dynamics. The straight-line distance measure was used to create the ‘pharmacy within a 20-minute walk’ variable. If the distance was less than 1.6 km (1 mile) then the variable was coded as yes.

Descriptive statistics were extracted for the outcomes availability per 10 000 people, distance to the nearest pharmacy and whether there was a pharmacy within a 20-minute walk. The descriptive statistics were stratified by IMD and urban/rural classification. All MSOAs had complete information on population, IMD and urban/rural classification, so no missing data treatment was needed.

Logistic regression was used to explore the association between the change over time in pharmacy availability and deprivation. We controlled for urban/rural classification, region, age structure (using median age) and ethnicity structure (proportion of people identifying as white).

### Patient and public involvement

No patients or members of the public were involved in the study.

## Results

### All England

**Figure 1:**
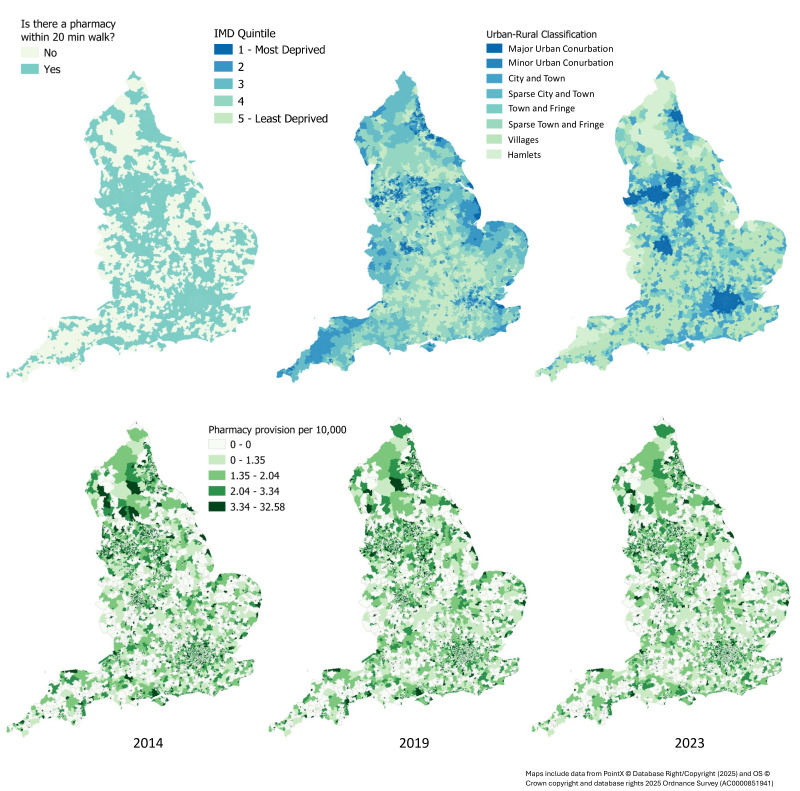
People living within a 20-minute walk to a community pharmacy by Index of Multiple Deprivation (IMD) and Urban Rural Classification (top) and community pharmacy availability per 10 000 people over time from 2014 to 2023 (bottom). Map 1 (top left) shows people living within a 20-minute walk of a community pharmacy. Blue means yes and light green means no. Map 2 (top middle) shows IMD quintile where blue is the most deprived and green is the least deprived. Map 3 (top right) shows urban/rural classification where blue is most urban, and green is most rural. The bottom maps show pharmacy provision per 10 000 people where the darker green means higher provision. From left to right is data from 2014, 2019 and 2023.

**Figure 2:**
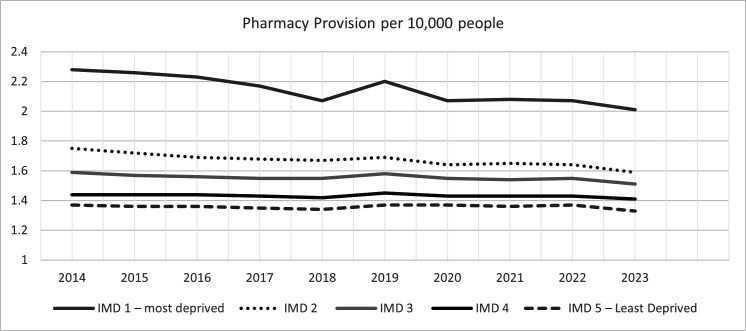
Community pharmacy availability per 10 000 people between 2014 and 2023 by Index of Multiple Deprivation (IMD) quintile. This figure shows a line graph with year on the x-axis and provision per 10 000 people on the y-axis. IMD 1 is dark grey solid line, IMD 2 is dark grey dotted line, IMD 3 is light grey solid line, IMD 4 is black solid line and IMD 5 is dark grey dashed line.

Overall, the analysis showed that, in 2014, 91.3% of people lived within a 20-minute walk to a community pharmacy and, in 2023, this number is 91.7%, showing that this measure has remained stable. The areas in England with a community pharmacy within a 20-minute walk in 2023 are shown visually in figure 1, alongside community pharmacy distribution by IMD and urban/rural classification. The values for community pharmacy availability per 10 000 people and spatial accessibility from years 2014 to 2023 are shown in online supplemental tables S3 and S4. Across England, the median number of community pharmacies per 10 000 people in 2014 was 1.60 (1.57, 1.64) while in 2023, the number reduced to 1.51 (1.49, 1.54) community pharmacies per 10 000 people, showing an overall decline of 0.09 of community pharmacies per 10 000 (a reduction of community pharmacies per population by 5.6%). The availability of community pharmacies per 10 000 people over time is shown visually in figure 2.

### Availability and access to community pharmacy by IMD quintile

**Figure 3:**
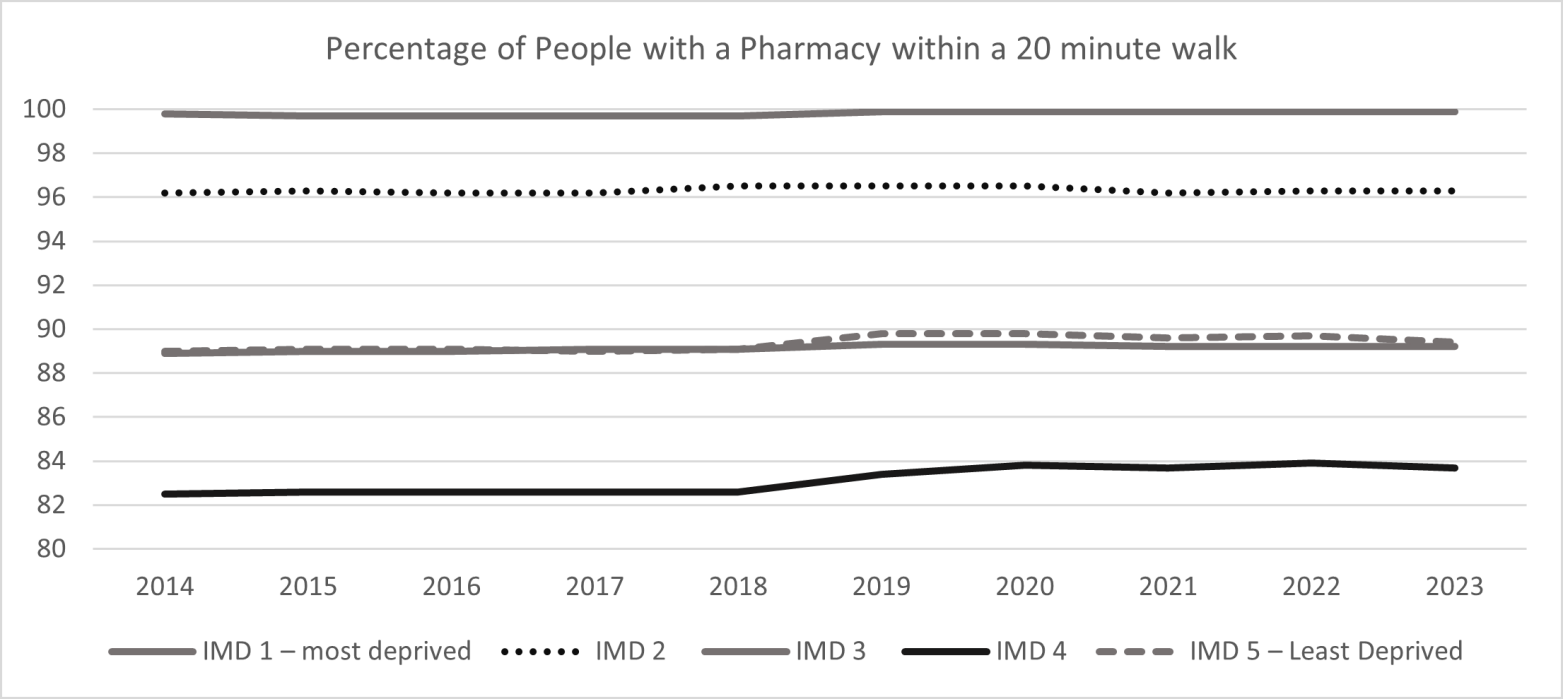
Community pharmacy accessibility within 20-minute walking distance between 2014 and 2023 by Index of Multiple Deprivation (IMD) quintile. This figure shows a line graph with year on the x-axis and percentage of people with access within 20-minute walking distance on the y-axis. IMD 1 is dark grey solid line, IMD 2 is dark grey dotted line, IMD 3 is light grey solid line, IMD 4 is black solid line and IMD 5 is dark grey dashed line.

There was a positive relationship between community pharmacy availability and deprivation—the more deprived an area, the higher the community pharmacy availability (table 1 and figure 2). In 2014, there were 2.28 (2.12, 2.39) pharmacies per 10 000 people in the 20% most deprived areas and 1.37 (1.34, 1.44) in the 20% least deprived areas. In 2023, this availability was reduced to 2.01 (1.87, 2.17) pharmacies per 10 000 people in the 20% most deprived areas and 1.33 (1.29, 1.38) in the 20% least deprived areas. Over the study period, community pharmacy availability reduced across all deprivation quintiles, with the sharpest decline in the 20% most deprived areas (−0.27/11.8%), which was around four times the decline of availability seen in the 20% least deprived areas (−0.04/3%). Accessibility of community pharmacy by spatial access showed that, in 2014, for the most deprived 20% areas, 99.8% of people could access a community pharmacy within a 20-minute walk, compared with 89.0% for the least deprived 20% areas. There was an observed U-shaped relationship between spatial access to a community pharmacy and deprivation, whereby people living in the 3rd and 4th quintiles had 88.9% and 82.5% access, respectively. The percentages of people who could access a community pharmacy within a 20-minute walk have remained stable over time and by IMD quintile (table 2 and figure 3).

**Table 1:** Community pharmacies per 10 000 persons by IMD quintile, median

Year	IMD 1—most deprived	IMD 2	IMD 3	IMD 4	IMD 5—least deprived
2014	2.28	1.75	1.59	1.44	1.37
2015	2.26	1.72	1.57	1.44	1.36
2016	2.23	1.69	1.56	1.44	1.36
2017	2.17	1.68	1.55	1.43	1.35
2018	2.07	1.67	1.55	1.42	1.34
2019	2.2	1.69	1.58	1.45	1.37
2020	2.07	1.64	1.55	1.43	1.37
2021	2.08	1.65	1.54	1.43	1.36
2022	2.07	1.64	1.55	1.43	1.37
2023	2.01	1.59	1.51	1.41	1.33
Total change (2014–2023)	−0.27 (11.8%)	−0.16 (9%)	−0.08 (5%)	−0.03 (2%)	−0.04 (3%)

IMD, Index of Multiple Deprivation.

**Table 2:** Percentage of people with a community pharmacy in 1 mile walk (%) per year by IMD

Year	IMD 1—most deprived		IMD 2		IMD 3		IMD 4		IMD 5—least deprived	
	Yes	No	Yes	No	Yes	No	Yes	No	Yes	No
2014	99.8	0.2	96.2	3.8	88.9	11.1	82.5	17.5	89.0	11.0
2015	99.7	0.3	96.3	3.7	89.0	11.0	82.6	17.4	89.1	10.9
2016	99.7	0.3	96.2	3.8	89.0	11.0	82.6	17.4	89.1	10.9
2017	99.7	0.3	96.2	3.8	89.1	10.9	82.6	17.4	89.0	11.0
2018	99.7	0.3	96.5	3.5	89.1	10.9	82.6	17.4	89.1	10.9
2019	99.9	0.1	96.5	3.5	89.3	10.7	83.4	16.6	89.8	10.2
2020	99.9	0.1	96.5	3.5	89.3	10.7	83.8	16.2	89.8	10.2
2021	99.9	0.1	96.2	3.8	89.2	10.8	83.7	16.3	89.6	10.4
2022	99.9	0.1	96.3	3.7	89.2	10.8	83.9	16.1	89.7	10.3
2023	99.9	0.1	96.3	3.7	89.2	10.8	83.7	16.3	89.4	10.6

IMD, Index of Multiple Deprivation.

### Urban/rural availability and community pharmacy access

**Figure 4:**
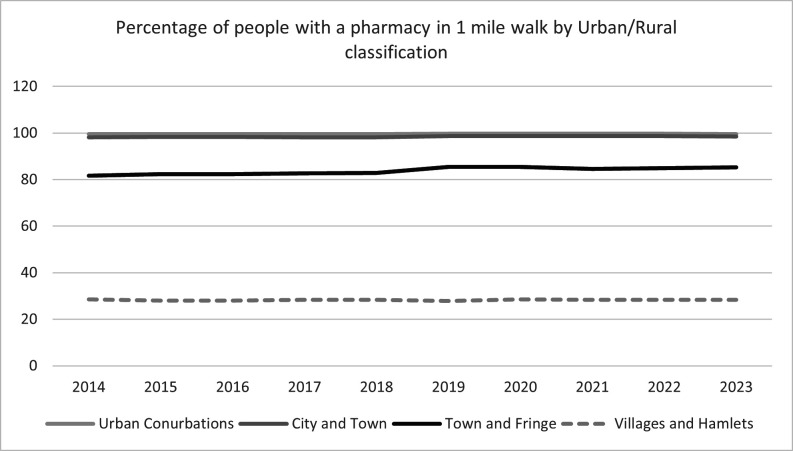
Percentage of people with a pharmacy within a 20-minute walk between 2014 and 2023 by urban/rural classification. This figure shows a line graph with year on the x-axis and percentage of people with access within 20-minute walking distance on the y-axis. Urban conurbations are light grey solid line, city and town is light grey solid line, town and fringe is black solid line and villages and hamlets are light grey dashed line.

In major and minor urban conurbations, the median community pharmacy availability was 1.81 (1.70, 1.94) pharmacies per 10 000 people in 2014 and 1.66 (1.60, 1.74) per 10 000 people in 2023, showing a decline of 0.15 pharmacies per 10 000 people or a reduction by 8.2%. In cities and towns, and towns and fringe, the median pharmacy availabilities were similar, starting at 1.66 (1.61, 1.71) and 1.64 (1.54, 1.70) in 2014 and reducing to 1.54 (1.49, 1.58) and 1.56 (1.51, 1.62), respectively, by 2023. In villages and hamlets, the median community pharmacy availability was zero, which can be interpreted as over 50% of areas classified as villages and hamlets have no pharmacy within the MSOA (table 3). Considering spatial access to a community pharmacy, for urban conurbations, access remained constant over time: in 2014, the access was 99.6% for major and minor urban conurbations and 98.2% for towns and cities, while in 2023, the access was 99.6% and 98.5% for major and minor urban conurbations and towns and cities, respectively. For towns and fringe, 81.7% of people had a community pharmacy within a 20-minute walk in 2014, which increased to 85.2% in 2023. In contrast, in 2014 only 28.3% of people in villages and hamlets had a community pharmacy within a 20-minute walk, which remained relatively constant over time to 2023 (online supplemental table S5 and figure 4).

**Table 3:** Community pharmacies per 10 000 persons by urban/rural category, median

Year	Major and minor urban conurbations	Cities and towns	Towns and fringe	Villages and hamlets
2014	1.81	1.66	1.64	0
2015	1.78	1.64	1.64	0
2016	1.76	1.62	1.63	0
2017	1.73	1.63	1.62	0
2018	1.70	1.59	1.59	0
2019	1.79	1.62	1.62	0
2020	1.70	1.59	1.57	0
2021	1.70	1.58	1.57	0
2022	1.70	1.59	1.57	0
2023	1.66	1.54	1.56	0
Total change	−0.15	−0.12	−0.08	0

There is a positive relationship between urbanicity and deprivation, whereby as urbanicity increases, deprivation also increases (online supplemental table S2). Overall, deprived areas have higher availability of community pharmacies per 10 000 people and have greater spatial access to community pharmacies across all urban/rural classifications (online supplemental table S6). For example, in the most rural deprived areas, 50% of people have access to a community pharmacy within a 20-minute walk, and there are 2.08 community pharmacies per 10 000 people. In the least deprived rural areas, only 32.2% of people have a community pharmacy within a 20-minute walk and there are a median of zero community pharmacies within the MSOA.

### Change over time and the association with deprivation

Table 4 shows the results of the logistic regression which explored the association between the loss of pharmacy availability per 10 000 people and area level deprivation. The model was adjusted for age structure, ethnicity structure, urban/rural classification and region.

**Table 4:** Logistic regression showing unadjusted and adjusted ORs for the association between loss of a pharmacy and deprivation quintile

Change between 2014 and 2023	OR	SE	t	P>t	(95% CI)
Unadjusted					
IMD quintile (relative to 5; least deprived quintile)					
4	1.06	0.08	0.77	0.44	(0.91, 1.23)
3	1.15	0.09	1.82	0.07	(0.99, 1.34)
2	1.40	0.11	4.33	0.00	(1.20, 1.63)
1 (most deprived)	1.79	0.14	7.40	0.00	(1.53, 2.08)
Adjusted					
IMD quintile (relative to 5; least deprived quintile)					
4	1.11	0.09	1.31	0.19	(0.95, 1.29)
3	1.16	0.09	1.86	0.06	(0.99, 1.35)
2	1.31	0.11	3.27	0.00	(1.12, 1.54)
1 (most deprived)	1.65	0.15	5.45	0.00	(1.38, 1.98)
Urban/rural (relative to major and minor urban conurbations)					
City and town	1.03	0.06	0.48	0.63	(0.91, 1.17)
Town and fringe	1.88	0.19	6.07	0.00	(1.53, 2.30)
Villages	0.63	0.07	−4.31	0.00	(0.51, 0.78)
Region (relative to north)					
Midlands	1.25	0.09	2.91	0.00	(1.07, 1.45)
South	1.31	0.09	4.06	0.00	(1.15, 1.49)
Median age	0.97	0.01	−5.10	0.00	(0.96, 0.98)
Proportion of white people	1.25	0.26	1.07	0.28	(0.83, 1.87)

After adjustment, compared with the 20% least deprived areas, the 20% most deprived areas (IMD quintile 1) were more likely to lose a pharmacy (OR 1.65 (1.38, 1.98)). IMD quintile 2 was also more likely to lose a pharmacy (OR 1.31 (1.12, 1.54), compared with the least deprived quintile (IMD quintile 5). There was no effect in the less deprived quintiles (IMD quintiles 3 and 4). Sensitivity analysis (in online supplemental tables S9 and S10) shows that the results are robust to operationalising the outcome variable in a different way and using linear regression.

## Discussion

This paper identifies several important findings: first, there is high access to community pharmacies in all areas of England, with over 90% of the population living within a 20-minute walk of a community pharmacy; second, the ‘positive pharmacy care law’ still stands 10 years on as there is greatest access to community pharmacies in the most deprived and urban areas; and finally, even though geographical access has remained constant, the reduction in the number of community pharmacies in England means that each community pharmacy serves a higher number of people, with the greatest decline in availability in the most deprived areas. The 20% most deprived areas experienced a decrease from 2.28 pharmacies per 10 000 people in 2014 to 2.01 pharmacies per 10 000 people in 2023 and were more likely to lose a pharmacy (OR 1.65 (1.38, 1.98)) when compared with the least deprived areas.

### Policy implications

The work shows that the ‘positive pharmacy care law’ is still in existence, although it appears to be eroding, given that the availability of community pharmacies has reduced—particularly in deprived areas where more people are served by community pharmacies due to a lack of other healthcare options. Commissioning healthcare and public health services through community pharmacies still has scope to reach people in the greatest need due to having more community pharmacies in deprived areas. In view of this, the development of new community pharmacy services, particularly those designed to increase access to tackle inequalities in health outcomes, is warranted. Good examples in this regard are the current services of influenza and COVID-19 vaccinations or services to promote early cancer detection through screening and direct referral interventions.^31 32^

There is now a greater reliance on community pharmacies per 10 000 population, and this is greatest in deprived areas—this puts pressure on services and staff. Indeed, due to the nature of the National Health Service (NHS) Community Pharmacy Contractual Framework in England and the tiered levels of services, there is potential that there will be less capacity to provide the additional enhanced clinical services for community pharmacies located in the most deprived areas. For example, dispensing medication is considered a core ‘essential’ service that must be provided by all community pharmacies while other services, such as smoking cessation, vaccination and hypertension screening are additional services that can be offered by the community pharmacy, providing there is sufficient resource (staff to deliver the services and infrastructure to provide the services). Reviewing the NHS Community Pharmacy Contractual Framework in England to ensure there is no tension between delivering core essential services and enhanced clinical services appears to be warranted.

Community pharmacies have recently highlighted workforce challenges and staff shortages, which have been linked to a lack of funding.^33^ This lack of funding has also seen the closure of multiple community pharmacies in England. Indeed, there has been debate about the challenges of community pharmacy funding, with the sector in England recently holding a national day of protest.^34^ These disputes are also mirrored across Europe, with pharmacists in France recently holding a national strike day in relation to the challenges associated with funding.^35^ If the policy directives of allowing community pharmacies to deliver more clinical services to support other primary care organisations are to reach their full potential, it is important that appropriate funding arrangements are in place to achieve this.

### Strengths and weaknesses

In this study, we conceptualised access to a community pharmacy in two ways: first, the pharmacy availability in a person’s ‘local area’ and second, the distance to the closest pharmacy for most of the population. To facilitate this, there are some methodological changes from our original 2014 study around the conceptualisation of ‘local area’ and the distance calculation to the community pharmacy. For this work, we used MSOAs rather than Lower layer Super Output Areas (LSOAs) to represent a person’s local area. We believe that the larger MSOAs are a better conceptual representation of a person’s local area or town than an LSOA, so if a community pharmacy is no longer in their MSOA, it means that access is more difficult since travelling to another MSOA may be perceived as leaving their local area.

To find the percentage of the population that can access a pharmacy, the 2014 study used a catchment area approach by creating a 1.6 km buffer around each pharmacy and calculating the percentage of the population covered by the buffer. Instead, we used a distance approach from the population-weighted centroid (usually located in the largest population centre) of the MSOA to the nearest pharmacy, and if the distance was less than 1.6 km, we concluded that the people of that MSOA have access to a community pharmacy within a 20-minute walk. This approach is conceptually very similar but less computationally difficult than a catchment area approach. As in the 2014 paper, we used a straight-line distance to conceptualise spatial access since this approach is less computationally intensive than street network distance. While this may be acknowledged as a limitation, previous work has shown that added accuracy from using street network distance has been found to be insignificant in most cases.^36 37^

The study also has some methodological limitations around data availability. The MSOA boundaries used in the analysis are from 2011 rather than the latest from 2021; this approach was used to enable the linkage of the latest IMD 2019 and 2011 urban/rural classification data. In addition, the pharmacy per 10 000 people measure uses the midyear population estimate from 2014 to 2020. For the years 2021–2023, we used the 2020 population estimate since more recent data were not available. This may lead to the overestimation of the community pharmacies per 10 000 people for years post 2020 as the real population denominator may have increased.

Due to being an area-level study, we acknowledge the limitation that the findings may not be directly applicable to individuals with different ages, comorbidities and behaviours. We also acknowledge that just because a person lives within 20-minute walking distance (1 mile) of a community pharmacy does not necessarily mean they can walk the distance; there could be a number of physical, mental and cultural factors that impact on an individual’s walking ability. However, the strength of area-level findings is that they can be used to inform country-wide availability of pharmacies and highlight differences in geographic contexts, over and above individual capabilities.

### Conclusion

There is high access to community pharmacies in England with access to a community pharmacy greatest in the most deprived areas, showing that the ‘positive pharmacy care law’ remains. However, the ‘positive pharmacy care law’ is eroding as the availability of community pharmacies has reduced over time—particularly in deprived areas, with more people reliant on each community pharmacy. Reinvestment in the community pharmacy network will help reduce inequalities in access to healthcare.

## Supplementary material

10.1136/bmjopen-2024-095540 online supplemental file 1

## Data Availability

Data are available upon reasonable request. Data may be obtained from a third party and are not publicly available.
