# Supplementary material for: The Positive Pharmacy Care Law revisited: an area-level analysis of the relationship between community pharmacy distribution, urbanicity and deprivation in England
Source: BMJ Open. 2025 May 11;15(5):e095540. doi: 10.1136/bmjopen-2024-095540 (PMC12067833; doi:10.1136/bmjopen-2024-095540)
Supplement: online supplemental file 1 [file bmjopen-15-5-s001.docx]

**Supplementary material**

**Relationship between Deprivation and urbanicity**

| Supplementary Table 1: Classification of 8 groups into 4 | | |
| --- | --- | --- |
| Code | Number | Classification |
| A1 | 2,399 | Major Urban Conurbations  Minor Urban Conurbations |
| B1 | 249 |  |
| C1 | 2,938 | City and Town  City and Town in Sparse Setting |
| C2 | 13 |  |
| D1 | 588 | Town and Fringe  Town and Fringe in Sparse Setting |
| D2 | 20 |  |
| E1 | 539 | Villages and Hamlets  Villages and Hamlets in Sparse Setting |
| E2 | 45 |  |

| Supplementary Table 2: Relationship between IMD and Urban/Rural Classification groups | | | | | | | | | | | | |
| --- | --- | --- | --- | --- | --- | --- | --- | --- | --- | --- | --- | --- |
|  | IMD 1 – most deprived | | 2 | | 3 | | 4 | | 5 | | Total |  |
|  | N | % | N | % | N | % | N | % | N | % |  |  |
| A1/B1 | 795 | 58.5 | 664 | 48.9 | 499 | 36.7 | 342 | 25.2 | 348 | 25.6 | 2648 | 39.0 |
| C1/C2 | 539 | 39.7 | 571 | 42.0 | 579 | 42.6 | 571 | 42.0 | 691 | 50.8 | 2951 | 43.5 |
| D1/D2 | 22 | 1.6 | 68 | 5.0 | 115 | 8.5 | 198 | 14.6 | 205 | 15.1 | 608 | 9.0 |
| E1/E2 | 2 | 0.1 | 55 | 4.1 | 165 | 12.2 | 247 | 18.2 | 115 | 8.5 | 584 | 8.6 |
| Total | 1358 |  | 1358 |  | 1358 |  | 1358 |  | 1359 |  | 6791 |  |

**Descriptive Statistics for All of England:**

| Supplementary Table 3: All of England Pharmacy availability | | | | |
| --- | --- | --- | --- | --- |
| Year | Total # open | MSOAs with at least 1 pharmacy | Median pharmacies per 10,000 people | Median distance to pharmacy |
| 2014 | 11776 | 5366 | 1.60 | 0.456 |
| 2015 | 11617 | 5369 | 1.59 | 0.454 |
| 2016 | 11453 | 5379 | 1.57 | 0.454 |
| 2017 | 11283 | 5386 | 1.56 | 0.454 |
| 2018 | 11249 | 5380 | 1.54 | 0.455 |
| 2019 | 11214 | 5451 | 1.58 | 0.447 |
| 2020 | 10749 | 5431 | 1.54 | 0.448 |
| 2021 | 10795 | 5427 | 1.54 | 0.452 |
| 2022 | 10749 | 5431 | 1.54 | 0.452 |
| 2023 | 10494 | 5388 | 1.51 | 0.454 |
| Total Change | -1,282/-10.8% | +22/+0.40% | -0.09/5.6% | +0.02 |

| Supplementary Table 4: Percentage of people with a Pharmacy in 1 mile walk in all of England | | |
| --- | --- | --- |
| Year | [% (N)] | |
|  | Yes | No |
| 2014 | 91.3 (6200) | 8.7 (591) |
| 2015 | 91.3 (6203) | 8.7 (588) |
| 2016 | 91.3 (6203) | 8.7 (588) |
| 2017 | 91.3 (6203) | 8.7 (588) |
| 2018 | 91.3 (6203) | 8.7 (588) |
| 2019 | 91.8 (6231) | 8.2 (560) |
| 2020 | 91.8 (6236) | 8.2 (555) |
| 2021 | 91.7 (6227) | 8.3 (564) |
| 2022 | 91.8 (6234) | 8.2 (557) |
| 2023 | 91.7 (6228) | 8.3 (563) |

| Supplementary Table 5: percentage of population with a pharmacy in 20 minute walk by urban/rural classification | | | | | | | | |
| --- | --- | --- | --- | --- | --- | --- | --- | --- |
| **Year** | **Major and Minor Urban Conurbations** | | **Cities and Towns** | | **Town and Fringe** | | **Villages and Hamlets** | |
|  | **Yes** | **No** | **Yes** | **No** | **Yes** | **No** | **Yes** | **No** |
| **2014** | 99.6 | 0.4 | 98.2 | 1.8 | 81.7 | 18.3 | 28.6 | 71.4 |
| **2015** | 99.6 | 0.4 | 98.3 | 1.7 | 82.4 | 17.6 | 28.1 | 71.9 |
| **2016** | 99.6 | 0.4 | 98.3 | 1.7 | 82.4 | 17.6 | 28.1 | 71.9 |
| **2017** | 99.6 | 0.4 | 98.2 | 1.8 | 82.7 | 17.3 | 28.3 | 71.7 |
| **2018** | 99.6 | 0.4 | 98.1 | 1.9 | 82.9 | 17.1 | 28.4 | 71.6 |
| **2019** | 99.7 | 0.3 | 98.6 | 1.4 | 85.4 | 14.6 | 27.9 | 72.1 |
| **2020** | 99.7 | 0.3 | 98.6 | 1.4 | 85.4 | 14.6 | 28.6 | 71.4 |
| **2021** | 99.7 | 0.3 | 98.6 | 1.4 | 84.5 | 15.5 | 28.3 | 71.7 |
| **2022** | 99.7 | 0.3 | 98.6 | 1.4 | 85.0 | 15.0 | 28.3 | 71.7 |
| **2023** | 99.6 | 0.4 | 98.5 | 1.5 | 85.2 | 14.8 | 28.3 | 71.7 |

| Supplementary Table 6: % Access (within 1 mile walk) in 2023 | | | | | |
| --- | --- | --- | --- | --- | --- |
| IMD Quintile | All England | Major and Minor Urban Conurbations | Cities and Towns | Town and Fringe | Villages and Hamlets |
| 1 – most deprived | 99.9 | 100.0 | 99.8 | 100.0 | 50.0 |
| 2 | 96.3 | 99.8 | 99.8 | 85.3 | 30.9 |
| 3 | 89.2 | 99.4 | 99.5 | 92.2 | 20.6 |
| 4 | 83.7 | 99.1 | 97.2 | 84.3 | 30.8 |
| 5 – least deprived | 89.4 | 99.1 | 96.7 | 80.5 | 32.2 |
| Median pharmacies per 10,000 in 2023 | | | | | |
| IMD Quintile | All England | Major and Minor Urban Conurbations | Cities and Towns | Town and Fringe | Villages and Hamlets |
| 1 – most deprived | 2.01 | 2.00 | 2.08 | 1.64 | 2.08 |
| 2 | 1.59 | 1.61 | 1.66 | 1.88 | 1.00 |
| 3 | 1.51 | 1.61 | 1.63 | 1.92 | 0 |
| 4 | 1.41 | 1.60 | 1.43 | 1.53 | 0 |
| 5 – least deprived | 1.33 | 1.49 | 1.32 | 1.41 | 0 |

# Pharmacies Data Cleaning Supplement

The aim of this section is to outline the data investigation and cleaning procedure for the Ordnance Survey Points of Interest dataset^1^ to harmonise it with the list of registered pharmacies by the General Pharmaceutical Service.^2^

**The Ordnance Survey Dataset**

This dataset covers the entire United Kingdom, so the first step was to filter the points of interest to those in England only. There were approximately 3 million points of interest per year in England. The points of interest are organised by a general category, for example, “Health and Education” then a further class name – in our case “Chemists and Pharmacies”. Each point of interest also has its name, spatial co-ordinates, address and data source registered in the dataset. We used the data from September of each year 2014-2023.

**General Pharmaceutical Services**

The General Pharmaceutical Services in England collects information about community pharmacy contractors and dispensing of prescription items. Pharmacy premises must be registered and reviewed every year, so the number of pharmacies in the dataset is the definitive number in England in each financial year. We excluded distance selling pharmacies since they are not necessarily physically accessible like a community pharmacy. The data is available for the financial year (April to April) from 2015/16 to 2022/23.

| Supplementary Table 7: Comparison between the Ordnance Survey Dataset and General Pharmaceutical Services Dataset | | | | | | |
| --- | --- | --- | --- | --- | --- | --- |
|  | Number of registered pharmacies in GPS excluding distance selling | | Number of pharmacies in the OS Dataset using “Chemists and Pharmacies” Class Name | | Difference between OS and GPS | |
| Year |  | Year on year change |  | Year on year change | N | % |
| **2014** |  |  | 10750 |  | - | - |
| **2015** |  |  | 10868 |  | - | - |
| **2016** | 11822 |  | 10858 |  | -964 | -8.2 |
| **2017** | 11586 | -236 | 10843 | -15 | -743 | -6.4 |
| **2018** | 11616 | +30 | 10715 | -128 | -901 | -7.8 |
| **2019** | 11511 | -105 | 11238 | +523 | -273 | -2.4 |
| **2020** | 11436 | -75 | 11508 | +270 | 72 | 0.6 |
| **2021** | 11264 | -172 | 11205 | -303 | -59 | -0.5 |
| **2022** | 11147 | -117 | 11085 | -120 | -62 | -0.6 |
| **2023** | 11016 | -131 | 10804 | -281 | -212 | -1.9 |

Table 7 illustrates the number of registered community pharmacies, and the number of pharmacies present in the OS dataset when using the “chemists and pharmacies” class name. We expected that there would be differences in the numbers in both datasets due to the difference in the data collection period (rolling financial year vs one snapshot in September). We also expected missing data in the OS dataset as they state that the dataset is not a complete record of all points of interest. While there is no absolute cut off, it is generally posited that around 5% missingness can be ignored in spatial datasets with minimal risk of bias.^3,4^

However, the issue here is not the overall percentage of missing data, rather the *pattern* of missingness and how it relates to the registered pharmacies in the GPS dataset. For the community pharmacies, there has been a steady decline since 2016 (barring a slight increase in 2018). In the Ordnance Survey dataset, there are an additional 523 and 270 pharmacies added in 2019 and 2020 respectively. There is also a significant undercount between 2016 and 2019 – the missing data percentage is inconsistent across years. More importantly, this does not reflect the year-on-year change as shown in the GPS dataset – there was no big increase of pharmacies between 2019 and 2022, rather the opposite.

We think there are two issues in the dataset:

1. There is an undercount between 2016 – 2018 – the pharmacies may be in the dataset but not under the “Chemists and Pharmacies” class name
2. Pharmacies were either added to the dataset or reclassified in 2019 – this does not reflect an actual increase in pharmacies in reality.

**Data cleaning approach**

The data cleaning and validation process has two main aims – firstly, to determine if there are pharmacies present in the dataset that are not counted, and to find the reason for the additional pharmacies from 2019 onwards. Once determined, the data cleaning process should be applied to each year’s dataset for consistency, even if the missing data percentage is below 5%.

1. **Finding the missing pharmacies in the dataset**

Firstly, we investigated whether some pharmacies are present in the data but are misclassified (not under the “chemists and pharmacies” class name) using SQL filtering in QGIS.

*"classname"='Chemists and Pharmacies' OR "name" ILIKE '%pharmacy%' OR "name" ILIKE '%chemist%'*

This code selects everything that is within the class name 'Chemists and Pharmacies’ or which has pharmacy or chemist in the name of the point of interest. In 2016 this returned 12055 records, almost 2000 additional records.
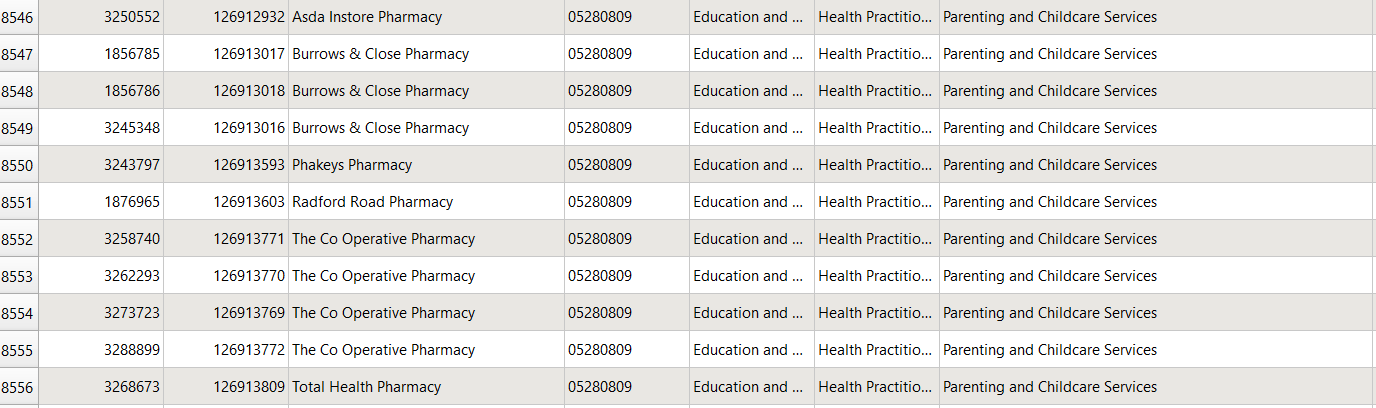
On closer inspection, we find that some pharmacies are misclassified – for example here as ‘Parenting and Childcare Services’


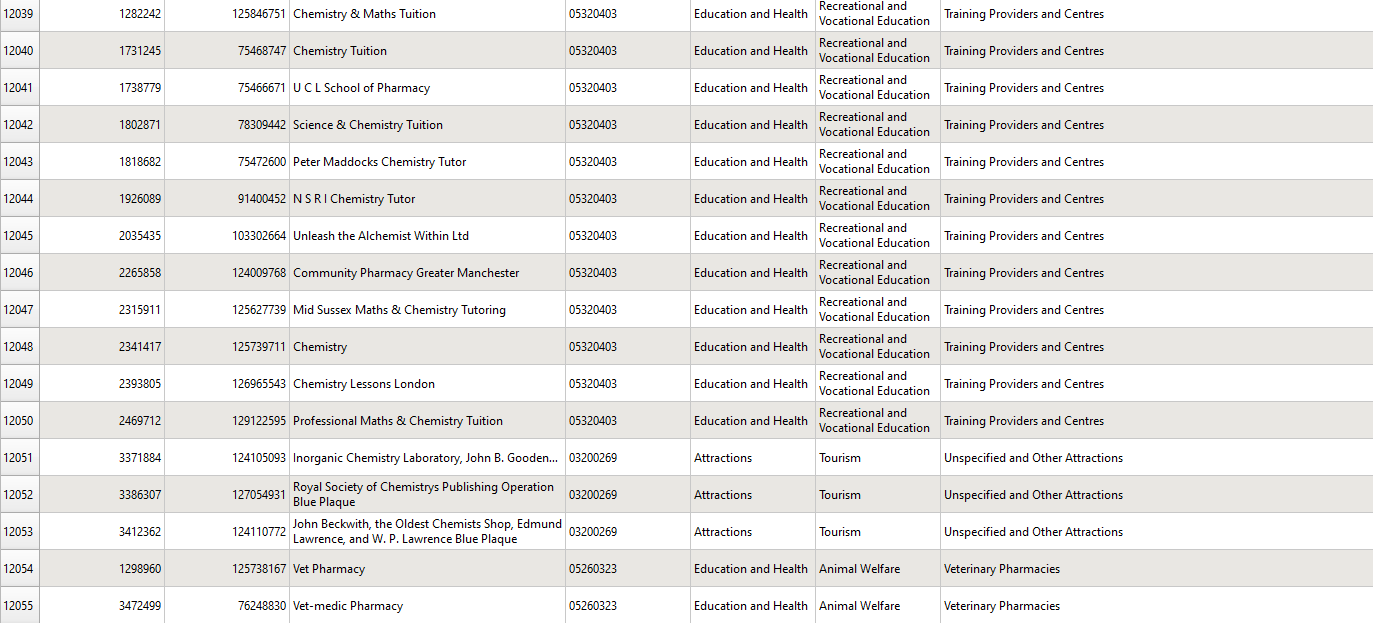
 We also find that some points of interest are clearly not community pharmacies, despite having pharmacy or chemist in the name:

Some of the points are duplicated in address or co-ordinates – or one premises has two functions (e.g., a pharmacy onsite and a cosmetics store) so has two entries


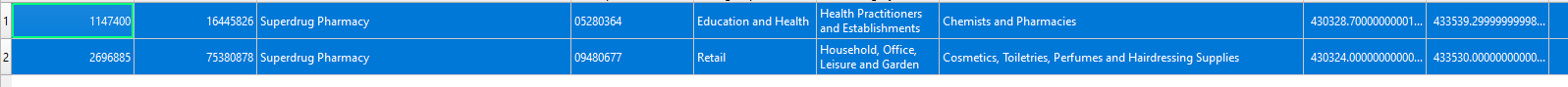
**Data Cleaning**

After selecting everything that might be a pharmacy, we manually filtered the results to remove entries that were likely not a pharmacy, for example, bus stops, cafes, or schools. We also removed duplicate geometries and exact addresses. We kept pharmacies classified under “Ambulance and Medical Transportation Services”, “Medical Equipment, Supplies and Pharmaceuticals” and “Cosmetics, Toiletries, Perfumes and Hairdressing Supplies” if there was Chemist/Pharmacy in the name.

1. **Reasons for additional pharmacies from 2019 onwards**

As outlined in table 7, there was a large increase of pharmacies in 2019 and 2020 which was not reflected in the GPS registered pharmacies dataset. To address this, we reached out to the Ordnance Survey Points of Interest team about the discrepancy. They informed us that after a data respecification in 2019 onwards *“additional NHS-run pharmacies which were found in hospitals and surgeries were added. This increased the number of chemist and pharmacies by around 600 records."* Additionally, after the 2019 respecification, there was more attention paid to misclassification and duplicates. We took this feedback into account and found that there were indeed pharmacies in hospitals included in the dataset which were not present before 2020, which we removed for years 2020-2023. We also found very few duplicates and misclassifications in the data from 2020 onwards, compared to 2014.

**Final Result**

| Supplementary Table 8 : Comparison between the Ordnance Survey Dataset and General Pharmaceutical Services Dataset after data cleaning | | | | | | |
| --- | --- | --- | --- | --- | --- | --- |
|  | Number of registered pharmacies in GPS excluding distance selling | | Number of pharmacies in the OS Dataset after cleaning and validation | | Difference between OS and GPS | |
| Year |  | Year on year change |  | Year on year change | N | % |
| **2014** |  |  | 11776 |  | - | - |
| **2015** |  |  | 11617 |  | - | - |
| **2016** | 11822 |  | 11453 |  |  |  |
| **2017** | 11586 | -236 | 11283 | -170 | -303 | 2.6% |
| **2018** | 11616 | +30 | 11249 | -34 | -367 | 3.1% |
| **2019** | 11511 | -105 | 11214 | -35 | -297 | 2.6% |
| **2020** | 11436 | -75 | 10749 | -465 | -687 | 6.0% |
| **2021** | 11264 | -172 | 10795 | +46 | -469 | 4.2% |
| **2022** | 11147 | -117 | 10749 | -46 | -398 | 3.6% |
| **2023** | 11016 | -131 | 10494 | -255 | -522 | 4.7% |
| **Total Change since 2016** | -806 |  | -789 |  |  |  |

The final count after cleaning shown in table 8 has addressed both issues of the undercount/missing data before 2019 and the overcount after 2019. The missing data is now between 2.6-6% which is a more acceptable percentage and trend than +0.6 to -8.2% missingness. We believe this approach is robust as we dealt with the missing data in a systematic way year on year and we consulted with the Ordnance Survey on their treatment of the data. The year on year change and total change is now closer to the trend shown by the registered pharmacies in the GPS dataset.

**References**

1. Ordnance Survey. OS MasterMap Points of Interest. (2014).

2. NHS Business Services Authority. General Pharmaceutical Services in England 2015/16 - 2022/23. (2023).

3. Butler, A. B., Kevin A. Best Practices for Dealing with Missing Data. *Esri* https://www.esri.com/about/newsroom/arcuser/dealing-with-missing-data/ (2019).

4. Montelpare, W. J., Read, E., McComber, T., Mahar, A. & Ritchie, K. Working with Missing Data. in *Applied Statistics in Healthcare Research* (2020).

# Sensitivity Analysis

In these models, the outcome measure of availability per 10,000 people was operationalised as continuous for two time points instead of change over time – the difference between the two time points. Two linear models were estimated for 2014 and 2023 respectively. The models were adjusted for urban/rural classification, region, age structure and ethnicity structure.

Unadjusted:

| Supplementary Table 9: Unadjusted Linear Models | | | | |
| --- | --- | --- | --- | --- |
|  | 2014 | | 2023 | |
| IMD Quintile | Co-efficient | 95% CI | Co-efficient | 95% CI |
| 4 | 0.18 | [0.03, 0.33] | 0.11 | [0.00, 0.23] |
| 3 | 0.44 | [0.29, 0.59] | 0.33 | [0.22, 0.45] |
| 2 | 0.69 | [0.54, 0.84] | 0.49 | [0.38, 0.61] |
| 1 (most deprived) | 1.23 | [1.08, 1.38] | 0.94 | [0.83, 1.06] |

Adjusted:

| Supplementary Table 10: Unadjusted Linear Models | | | | |
| --- | --- | --- | --- | --- |
|  | 2014 | | 2023 | |
| IMD Quintile | Co-efficient | 95% CI | Co-efficient | 95% CI |
| 4 | 0.30 | [0.15, 0.45] | 0.21 | [0.10, 0.33] |
| 3 | 0.46 | [0.31, 0.61] | 0.36 | [0.25, 0.48] |
| 2 | 0.56 | [0.40, 0.71] | 0.41 | [0.29, 0.53] |
| 1 (most deprived) | 1.00 | [0.82, 1.17] | 0.78 | [0.65, 0.91] |
|  |  |  |  |  |
| Urban/Rural |  |  |  |  |
| City and Town | 0.17 | [0.05, 0.29] | 0.05 | [-0.04, 0.14] |
| Town and Fringe | -0.05 | [-0.24, 0.14] | 0.03 | [-0.11, 0.14] |
| Villages | -1.19 | [-1.39, -0.99] | -1.06 | [-1.21, 0.14] |
|  |  |  |  |  |
| Region |  |  |  |  |
| Midlands | -0.17 | [-0.31, -0.03] | -0.19 | [-0.29, -0.08] |
| South | -0.18 | [-0.30, -0.06] | -0.24 | [-0.33, -0.15] |
|  |  |  |  |  |
| Median Age | 0.00 | [-0.01, 0.01] | 0.01 | [0.00, 0.02] |
| Proportion of white people | -0.42 | [-0.80, -0.04] | -0.56 | [-0.85, -0.27] |

The adjusted model in Table 10 shows that in 2014, the 20% most deprived areas had the strongest relationship with number of pharmacies per 10,000 people (1.00 [0.82, 1.17]) when compared with the 20% least deprived areas. However, this relationship is weaker in 2023 (0.78 [0.65, 0.91]). The differences in coefficients in 2014 is 0.7 [0.67, 0.72], while in 2023 it is 0.57 [0.55, 0.59], indicating that the strong relationship between deprivation and number of pharmacies per 10,000 is eroding.
