# Supplementary material for: The Positive Pharmacy Care Law revisited: an area-level analysis of the relationship between community pharmacy distribution, urbanicity and deprivation in England
Source: BMJ Open. 2025 May 11;15(5):e095540. doi: 10.1136/bmjopen-2024-095540 (PMC12067833; doi:10.1136/bmjopen-2024-095540)
Supplement: Supplementary file 2 [file bmjopen-2024-095540.reviewer_comments.pdf]

PEER REVIEW HISTORY

BMJ Open publishes all reviews undertaken for accepted manuscripts. Reviewers are asked to complete a checklist review form and are provided with free text boxes to elaborate on their assessment. These free text comments are reproduced below.

ARTICLE DETAILS

Title (Provisional)

The Positive Pharmacy Care Law Revisited: an area-level analysis of the relationship between community pharmacy distribution, urbanity and deprivation in England

Authors

Zied Abozied, Eman; Munford, Luke Aaron; Copeland, Alison; Kasim, Adetayo; Husband, Andy; Bambra, Clare; Todd, Adam

---

VERSION 1 - REVIEW

Reviewer 1

Name Rizk , John G

Affiliation Department of Practice, Sciences, and Health Outcomes Research, University of Maryland School of Pharmacy, Baltimore.

Date 21-Dec-2024

COI None

Thank you for the opportunity to review this work.

Introduction

• A clearer and more detailed definition of the “positive pharmacy care law” is needed. The current description does not fully explain its scope or implications.

• Line 52: The term "most deprived communities" requires further clarification. Are these communities those that are in the "most need"? If so, what is meant by "need"? Are these the economically poorest areas, or are they regions with a high prevalence of comorbidities? The current phrasing is unclear.

• Line 54: It would be helpful to explain what is meant by pharmacies being more “accessible.” Access is a multifaceted concept, and it is not solely determined by the number of pharmacies compared to primary care physicians. For example, one can walk into a pharmacy without an appointment, insurance is less of a concern, and there are no consultation fees. Therefore, it is important to clarify what is meant by "access" in this

context, especially in comparison to the rate of pharmacies per 1000 population relative to primary care physicians.

Methods

• How did the authors handle missing data? Did all MSOAs have complete information on population, IMD, and rurality? There is no mention of that.

• Did the authors control for age in their analysis? If an MSOA has a higher percentage of older adults, this could significantly influence the outcome, especially since it relates to walking distance. What about factors such as race/ethnicity?

• It is good to see that the authors clearly explain how they dealt with cleaning the pharmacy availability data, as I would expect this dataset to be less than perfect (as with most publicly available data).

• Is the term “provision” used in reference to population standard? I am more familiar with terms like "availability of" or "rate of [X] per [Y] population."

• The spatial analysis section could be rewritten for better clarity and understanding. The explanation of methodology here is somewhat complex and could be made more accessible.

Results

• The increase from 91.3% to 91.7% seems minimal, and it is unclear whether this represents a statistically significant change, especially given potential margin of error.

• The change in the median number of community pharmacies per 10,000 people from 1.6 to 1.5 between 2014 and 2023 is also not significant, in my opinion, given that the denominator (population size) is also likely changing. It would be useful to consider how population dynamics affect this figure.

• Has a sensitivity analysis been performed? For example, what about pharmacies located 20.5 minutes away (just slightly above the threshold)? It would be worth conducting a sensitivity analysis for this to assess whether there is a meaningful distinction between pharmacies that are 19.5 minutes away versus 20.5 minutes away.

• Overall, this appears to be a descriptive study, and the differences observed over a 10-year period are quite small, with changes in decimal points. Therefore, using terms like "increase" and "decrease" may not be the most accurate. I recommend exploring associations between factors rather than just describing the changes. Did the authors consider using logistic regression adjusted for potential confounders to investigate these associations more rigorously?

**Reviewer**	**2**
**Name**	**Hakariya, Hayase**

**Affiliation**	**Eberhard Karls University Tubingen Interfaculty Institute of Biochemistry**
**Date**	**10-Jan-2025**
**COI**	**None**

This authors performed unique study where they scrutinized geographical access to community pharmacies in England. While this effort should be appraised, I would have suggestions to the authors.

Major

- Overall, geographical community pharmacy access in England seems almost unchanged throughout the study period. The authors might consider showing how their result differ based on study settings i.e.) data cleaning condition or using another deprivation index, and so on. Such sensitivity analyses can show the consistency of the findings.
- In figure 2 (Table1), why didn't you show any standard deviation or 95% CI? Otherwise, we cannot know if you could say the pharmacy provision was indeed reduced or just within an error bar range.
- In the Discussion section, the authors could discuss not only policy implications for England, but also make cross-national comparisons based on literatures, or put the paper in the context of the findings of this i.e.) why you could see an increase in the percentage of people with a community pharmacy in 1 mile walk only in Town and Fringe area in Table 4.

Minor

- One might be interested in why global number of pharmacies in England has been decreasing so drastically.
- Introduction
    - Describing what kind of indications are majorly applied for these DOAC, and why you focused on the atrial fibrillation might help readers to comprehensively understand this manuscript.
- Method
    - P.6 line 12-: a bracket is maybe missing.
    - What is QGIS? It is not defined and might better to be briefly described at least what it stands for.
- Result
    - P.8 the last line: maybe typo. Figure1 seems to be Figure2.
    - Regarding Figure1, an elucidation of Urban-Rural Classification might help understand easily.
- Discussion
    - P.13 line 1-14: this part sounds exaggeration. In this study, how (deprived) individuals depend on community pharmacy hasn't investigated, and we cannot say that developing new community pharmacy services are warranted.

VERSION 1 - AUTHOR RESPONSE

**Reviewer 1:**	
A clearer and more detailed definition of the "positive pharmacy care law" is needed. The	Thank you for the comment. We have now expanded on the definition of the positive

current description does not fully explain its scope or implications.	pharmacy care law in the introduction to provide more information of the reader. We have also added the reference to the positive pharmacy care law paper, so it is referenced earlier in the introduction section.
Line 52: The term "most deprived communities" requires further clarification. Are these communities those that are in the "most need"? If so, what is meant by "need"? Are these the economically poorest areas, or are they regions with a high prevalence of comorbidities? The current phrasing is unclear.	We have clarified in text that 'most deprived' is based on area level deprivation. We have also added that highest need is based on an already existing high morbidity burden. The changes in text are:<br><br>"In 2014, we established that, in England, community pharmacies followed a 'positive pharmacy care law'[4] whereby the availability of community pharmacies was greatest in the most deprived communities (defined through area-level multiple deprivation). This was in contrast to the well-established 'inverse care law' for other health care services in England and internationally whereby there is less provision in the most deprived areas even though they have the highest health needs (i.e. a higher morbidity burden)"
Line 54: It would be helpful to explain what is meant by pharmacies being more "accessible." Access is a multifaceted concept, and it is not solely determined by the number of pharmacies compared to primary care physicians. For example, one can walk into a pharmacy without an appointment, insurance is less of a concern, and there are no consultation fees. Therefore, it is important to clarify what is meant by "access" in this context, especially in comparison to the rate of pharmacies per 1000 population relative to primary care physicians.	We have clarified that 'access' relates to spatial access i.e. living nearby to a pharmacy. We have added in text:<br><br>"In related analysis, we found that more people lived within a 20 minute walk to community pharmacies than other primary care providers, including general practice."
How did the authors handle missing data? Did all MSOAs have complete information on population, IMD, and rurality? There is no mention of that.	Thank you for highlighting this. There is no missing data in any of the MSOAs – we have added this in text:<br><br>"All MSOAs had complete information on population, IMD and urban/rural classification, so no missing data treatment was needed."

Did the authors control for age in their analysis? If an MSOA has a higher percentage of older adults, this could significantly influence the outcome, especially since it relates to walking distance. What about factors such as race/ethnicity?	We have adjusted for the age and ethnicity structure in the logistic regression exploring the change over time outcome of interest. The change in text is:<br><br>“Logistic regression was used to explore the association between the change over time in pharmacy availability and deprivation. We controlled for urban/rural classification, region, age structure (using median age) and ethnicity structure (proportion of people identifying as white).”<br><br>The distance is a spatial calculation rather than relating to individual capacities, we have highlighted this in the discussion:<br><br>“Due to being an area level study , we acknowledge the limitation that the findings may not be directly applicable to individuals with different ages, comorbidities and behaviours. We also acknowledge that just because a person lives within 20 minute walking distance (1 mile) of a community pharmacy does not necessarily mean they can walk the distance; there could be a number of physical, mental and cultural factors that impact on an individual’s walking ability. However, the strength of area level findings is that they can be used to inform country-wide availability of pharmacies and highlight differences in geographic contexts, over and above individual capabilities.”
It is good to see that the authors clearly explain how they dealt with cleaning the pharmacy availability data, as I would expect this dataset to be less than perfect (as with most publicly available data).	Thank you!
Is the term “provision” used in reference to population standard? I am more familiar with terms like "availability of" or "rate of [X] per [Y] population."	Thanks, we have changed this to ‘availability of’ as highlighted in yellow in the manuscript
The spatial analysis section could be rewritten for better clarity and understanding. The	We have included a more accessible terminology, but kept in the names of the

explanation of methodology here is somewhat complex and could be made more accessible.	algorithms if a reader wishes to replicate the methods:<br><br>"Two spatial analyses were undertaken in QGIS25 for each year of the dataset from 2014-2023:<br><br>1. The algorithm "count points in polygon" to find the number of pharmacies (represented as points) within the MSOA boundary polygon.<br><br>2. Straight line distance from population weighted centroid (the area where most people live) to the nearest pharmacy"
The increase from 91.3% to 91.7% seems minimal, and it is unclear whether this represents a statistically significant change, especially given potential margin of error.	Thank you for the comment - yes, we agree, we have highlighted that this is not a large increase and that the measure has remained stable over time.<br><br>"Overall, the analysis showed that, in 2014, 91.3% of people lived within a 20-minute walk to a community pharmacy and, in 2023, this number is 91.7%, showing that this measure has remained stable."
The change in the median number of community pharmacies per 10,000 people from 1.6 to 1.5 between 2014 and 2023 is also not significant, in my opinion, given that the denominator (population size) is also likely changing. It would be useful to consider how population dynamics affect this figure.	The population denominator also changes with each year, so the median reflects the growth of the population of that MSOA, except in the years 2021-2023 where data isn't available – we have clarified this in text:<br><br>"The number of community pharmacies in an MSOA was divided by the population of that MSOA to determine the availability per 10,000 people. The population denominator changes year on year to reflect population dynamics."
Has a sensitivity analysis been performed? For example, what about pharmacies located 20.5 minutes away (just slightly above the threshold)? It would be worth conducting a sensitivity analysis for this to assess whether there is a meaningful distinction between pharmacies that are 19.5 minutes away versus 20.5 minutes away.	We have conducted a sensitivity analysis for the change over time measure which is included in the appendix under 'Sensitivity Analysis'. We did not conduct further analysis on the walking distance measure as it did not change significantly over time.

Overall, this appears to be a descriptive study, and the differences observed over a 10-year period are quite small, with changes in decimal points. Therefore, using terms like "increase" and "decrease" may not be the most accurate. I recommend exploring associations between factors rather than just describing the changes. Did the authors consider using logistic regression adjusted for potential confounders to investigate these associations more rigorously?	Thank you for this great suggestion, we have now conducted a logistic regression using the availability per 10,000 people measure which changes over time. We investigated if a loss of a pharmacy was associated with area level deprivation. We adjusted for age structure, ethnicity structure, region and urban/rural classification.<br><br>The results are shown and discussed under the heading “Change over time and the association with deprivation” and presented in full in Table 4.<br><br>In the results we have added: “Table 4 shows the results of the logistic regression which explored the association between the loss of pharmacy availability per 10,000 people and area level deprivation. The model was adjusted for age structure, ethnicity structure, urban/rural classification and region.<br><br>Compared to the 20% least deprived areas, the 20% most deprived areas (IMD quintile 1) were more likely to lose a pharmacy (OR 1.65 [1.38, 1.98]). IMD quintile 2 were also more likely to lose a pharmacy (OR 1.31 [1.12, 1.54]. There was no effect in the less deprived quintiles (IMD quintiles 3 and 4) compared to the least deprived quintile (IMD quintile 5. Sensitivity analysis (in supplementary material) shows that the results are robust to operationalising the outcome variable in a different way and using linear regression.”<br><br>In the discussion we have added:<br><br>“The 20% most deprived areas experienced a decrease from 2.28 pharmacies per 10,000 people in 2014 to 2.01 pharmacies per 10,000 people in 2023 and were more likely to lose a pharmacy (OR 1.65 [1.38, 1.98]) when compared to the least deprived areas.”
**Reviewer 2:**	

These authors performed a unique study where they scrutinized geographical access to community pharmacies in England. While this effort should be appraised, I would have suggestions for the authors.	Thank you!
Overall, geographical community pharmacy access in England seems almost unchanged throughout the study period. The authors might consider showing how their results differ based on study settings i.e.) data cleaning condition or using another deprivation index, and so on. Such sensitivity analyses can show the consistency of the findings.	Thank you for this suggestion. Unfortunately, there are no data available to conduct such geographic sensitivity analysis – the IMD we use is the most up to date measure of deprivation available on MSOA level. The yearly data for pharmacy postcodes is also not available, only the aggregate data by region.<br><br>We have conducted a sensitivity analysis for the logistic regression we have added looking at the change over time in availability and its relationship with deprivation.
In figure 2 (Table1), why didn't you show any standard deviation or 95% CI? Otherwise, we cannot know if you could say the pharmacy provision was indeed reduced or just within an error bar range.	Thank you for the suggestion. We have now added in 95% CI to the reported medians in the results text:<br><br>e.g. "In 2014, there were 2.28 [2.12, 2.39] pharmacies per 10,000 people in the 20% most deprived areas…"<br><br>"In 2023, this availability was reduced to 2.01 [1.87, 2.17] pharmacies per 10,000 people in the 20% most deprived areas…"<br><br>This shows that the point estimate for 2023 is not contained within the CI for 2014.<br><br>We did not add them to the table itself since it impacted readability significantly – text became too small and cramped.
One might be interested in why global number of pharmacies in England has been decreasing so drastically.	Thank you for comment. We have now added a sentence in the discussion to explain that the lack of funding has led to a number of pharmacy closures.
Describing what kind of indications are majorly applied for these DOAC, and why you focused on the atrial fibrillation might help readers to comprehensively understand this manuscript.	We do not mention atrial fibrillation in the paper.

P.6 line 12-: a bracket is maybe missing.	Thanks, we have added this
What is QGIS? It is not defined and might better to be briefly described at least what it stands for.	We have added this text:<br><br>“The MSOA boundaries were linked to IMD, urban/rural classification, region and MSOA population estimate in Quantum Geographic Information System (QGIS) Software using a spatial join by MSOA ID code.”
P.8 the last line: maybe typo. Figure1 seems to be Figure2.	Thanks, we have edited this.
Regarding Figure1, an elucidation of Urban-Rural Classification might help understand easily.	Thank you, we have changed the figure legend for clarity.
o P.13 line 1-14: this part sounds exaggeration. In this study, how (deprived) individuals depend on community pharmacy hasn’t been investigated, and we cannot say that developing new community pharmacy services is warranted.	We have added that this is a policy implication:<br><br>“given the availability of community pharmacies has reduced – particularly in deprived areas where more people are served by community pharmacies due to a lack of other healthcare options. Commissioning healthcare and public health services through community pharmacies still has scope to reach people in the greatest need due to having more community pharmacies in deprived areas”

VERSION 2 - REVIEW

Reviewer 2

Name Hakariya, Hayase

Affiliation Eberhard Karls University Tubingen Interfaculty Institute of Biochemistry

Date 19-Mar-2025

COI

The authors addressed the reviewers' query adequately, and the revised manuscript is now suited for publication. The reviewer appreciates the authors' effort.
